# Supplementary material for: Quality of Life and Psychological Distress Related to Fertility and Pregnancy in AYAs Treated for Gynecological Cancer: A Systematic Review
Source: Cancers (Basel). 2024 Oct 12;16(20):3456. doi: 10.3390/cancers16203456 (PMC11506014; doi:10.3390/cancers16203456)
Supplement: Supplementary file 1 [file cancers-16-03456-s001.zip › cancers-3226902-supplementary.pdf]

Supplementary S1.

Literature searches

The following search strategies have been carefully conducted and tested by two researchers with knowledge of the subject, together with two librarians with expertise in scientific research and search methods.

Pubmed library

("Genital Neoplasms, Female"[majr] OR (("Neoplasms"[majr] OR "Neoplas*"[ti] OR "Tumor*"[ti] OR "Tumour*"[ti] OR "Cancer*"[ti] OR "malignan*"[ti] OR "oncolog*"[ti] OR "carcinoma*"[ti] OR "adenoma*"[ti] OR "Medical Oncology"[majr]) AND ("Gynecologic*"[ti] OR "Gynaecologic*"[ti] OR **"Female"[ti]** OR "uterine"[ti] OR "uterus"[ti] OR "endometri*"[ti] OR "cervix"[ti] OR "cervical"[ti] OR "ovar*"[ti] OR "vagina*"[ti] OR "vulva*"[ti] OR "fallopian"[ti]))) AND ("Therapeutics"[Mesh] OR "Therap*"[tw] OR "treat*"[tw] OR "Surgical Procedures, Operative"[Mesh] OR "surgical"[tw] OR "surgery"[tw] OR "operat*"[tw]) AND ("quality of life"[MeSH Terms] OR "quality of life"[Tw] OR QoL[Tw] OR "quality-of-life" OR "life quality"[Tw] OR "HRQOL"[tw] OR "distress*"[tw] OR "stress"[tw] OR "Stress, Psychological"[Mesh] OR "concerns"[tw] OR "regret*"[tw] OR "worrie*"[tw] OR "worry*"[tw] OR "psychosocial"[tw] OR "psycho-social"[tw] OR "emotion*"[Tw]) AND ("Pregnancy"[Mesh] OR "Pregnan*"[tw] OR "fertil*"[tw] OR "infertil*"[tw] OR "Fertility"[Mesh] OR "Infertility"[Mesh] OR "conception"[tw] OR "conceiv*"[tw] OR "child bearing"[tw] OR "childbearing"[tw]) NOT ("mice"[ti] OR "mouse"[ti] OR "rat"[ti] OR "rats"[ti] OR "rabbit*"[ti] OR "polycystic ovary syndrome" OR "fibroids")
On 30-11-2023: 781 results

Web of science

NOT (TS=("Genital Neoplasms, Female" OR "Neoplasms" OR "Neoplas*" OR "Tumor*" OR "Tumour*" OR "Cancer*" OR "malignan*" OR "oncolog*" OR "carcinoma*" OR "adenoma*" OR "Medical Oncology") AND TS=("Gynecologic*" OR "Gynaecologic*" OR **"Female"** OR "uterine" OR "uterus" OR "endometri*" OR "cervix" OR "cervical" OR "ovar*" OR "vagina*" OR "vulva*" OR "fallopian") AND TS=("Therapeutics" OR "Therap*" OR "treat*" OR "surgical" OR "surgery" OR "operat*") AND TS=("quality of life" OR "quality of life" OR QoL OR "quality-of-life" OR "life quality" OR "HRQOL" OR "distress*" OR "stress" OR "concerns" OR "regret*" OR "worrie*" OR "worry*" OR "psychosocial" OR "psycho-social") AND TI=("Pregnan*" OR "fertil*" OR "infertil*" OR "conception" OR "conceiv*" OR "child bearing" OR "childbearing") NOT TS=("mice" OR "mouse" OR "rat" OR "rats" OR "rabbit*" "polycystic ovary syndrome" OR "fibroids"))
On 30-11-2023; 613 results

Cochrane trial database

("Neoplas*" OR "Tumor*" OR "Tumour*" OR "Cancer*" OR "malignan*" OR "oncolog*" OR "carcinoma*" OR "adenoma*"):ti,**ab,kw** AND ("Gynecologic*" OR "Gynaecologic*" OR **"Female"** OR "uterine" OR "uterus" OR "endometri*" OR "cervix" OR "cervical" OR "ovar*" OR "vagina*" OR "vulva*" OR "fallopian"):**ti,ab,kw** AND ("Therapeutics" OR "Therap*" OR "treat*" OR "Surgical Procedures" OR "surgical" OR "surgery" OR "operat*"):**ti,ab,kw** AND ("quality of life" OR "QoL" OR "quality-of-life" OR "life quality" OR "HRQOL" OR "distress*" OR "stress" OR "concerns" OR "regret*" OR "worrie*" OR "worry*" OR "psychosocial" OR "psycho-social"):**ti,ab,kw** AND ("Pregnancy" OR "Pregnan*" OR "fertil*" OR "infertil*" OR "Fertility" OR "Infertility" OR "conception" OR "conceiv*" OR "child bearing" OR "childbearing"):ti NOT ("mice" OR "mouse" OR "rat" OR "rats" OR "rabbit*" "polycystic ovary syndrome" OR "fibroids"):ti
On 30-11-2023, 9 results.

PsycINFO

TX ("Pregnan*" OR "fertil*" OR "infertil*" OR "conception" OR "conceiv*" OR "child bearing" OR "childbearing") AND TX ("Neoplas*" OR "Tumor*" OR "Tumour*" OR "Cancer*" OR "malignan*" OR "oncolog*" OR "carcinoma*" OR "adenoma*") AND TX ("Gynecologic*" OR "Gynaecologic*" OR "Female" OR "uterine" OR "uterus" OR "endometri*" OR "cervix" OR "cervical" OR "ovar*" OR "vagina*" OR "vulva*" OR "fallopian") AND TX ("Therapeutics" OR "Therap*" OR "treat* OR "surgical" OR "surgery" OR "operat*") AND TX ("quality of life" OR QoL OR "quality-of-life" OR "life quality" OR "HRQOL" OR "well being" OR "distress*" OR "stress" OR "anxiety" OR "concerns" OR "regret*" OR "worrie*" OR "worry*" OR "psychosocial*" OR "psycho-social") NOT TI ("mice" OR "mouse" OR "rat" OR "rats" OR "rabbit*" "polycystic ovary syndrome" OR "fibroids")
On 30-11-2023, 433 results

Supplementary Table S1-S4 MMAT assessments

Table. S1: MMAT Assessment - Qualitative studies

Study	Is the qualitative approach appropriated to address the research question?	Are the qualitative data collection methods adequate to address the research question?	Are the findings adequately derived from the data?	Is the interpretation of results sufficiently substantiated by data?	Is there coherence between qualitative data sources, collection, analysis and interpretation?
Bentsen 2023	Yes	Yes	Yes	Yes	Yes
Komatsu 2022	Yes	Yes	Yes	Yes	Yes
Standen 2020	Yes	Unknown	Yes	Yes	No
Carter 2007	Yes	Unknown	Yes	No	Unknown

Table S2: MMAT Assessment Quantitative (non-randomized) studies

Study	Are the participants representative for the target group?	Are the measurements appropriate regarding both the outcome and intervention (or exposure)?	Are there complete outcome data?	Are the confounders accounted for in the design and analysis?	During the study period, is the intervention administered as intended?
Wenzel 2005	Yes	Yes	Yes	No	Yes

Table S3: MMAT Assessment Quantitative (descriptive) studies

Study	Is the sampling strategy relevant to address the research question?	Is the sample representative of the target population?	Are the measurements appropriate?	Is the risk of non-response bias low?	Is the statistical analysis appropriate to answer the research question?
Carter 2010	Yes	No	Yes	Yes	No
Chan 2016	Yes	Yes	Yes	Yes	Yes
Chen 2022	Yes	Yes	Yes	Unknown	Yes
Sait 2011	Yes	Yes	No	Unknown	No
Sobota, Ozakinci 2018	Yes	Yes	Yes	No	Yes
Young 2019	Yes	Yes	Yes	Unknown	Yes
Mattsson 2018	No	Yes	Yes	Yes	Yes

Table S4: MMAT Assessment mixed methods studies

Study	Is there an adequate rationale for using a mixed methods design to address the research question?	Are the different components of the study effectively integrated to answer the research question?	Are the outputs of the integration of qualitative and quantitative components adequately interpreted?	Are divergences and inconsistencies between quantitative and qualitative results adequately addressed?	Do the different components of the study adhere to the quality criteria of each tradition of the methods involved?
Carter, Sonoda 2010	Yes	Unknown	Yes	No	Unknown
Schlossman 2023	Yes	No	Yes	Yes	Yes
